# Case report: Cytokine hemoadsorption in a case of hemophagocytic lymphohistiocytosis secondary to extranodal NK/T-cell lymphoma

**DOI:** 10.3389/fmed.2022.925751

**Published:** 2022-08-15

**Authors:** Juan Carlos Ruiz-Rodríguez, Luis Chiscano-Camón, Adolf Ruiz-Sanmartin, Clara Palmada, Ivan Bajaña, Gloria Iacoboni, Camilo Bonilla, Alejandra García-Roche, Erika Paola Plata-Menchaca, Carolina Maldonado, Marcos Pérez-Carrasco, Mónica Martinez-Gallo, Clara Franco-Jarava, Manuel Hernández-González, Ricard Ferrer

**Affiliations:** ^1^Intensive Care Department, Vall d'Hebron University Hospital, Vall d'Hebron Barcelona Hospital Campus, Barcelona, Spain; ^2^Shock, Organ Dysfunction and Resuscitation Research Group, Vall d'Hebron Research Institute (VHIR), Vall d'Hebron University Hospital, Vall d'Hebron Barcelona Hospital Campus, Barcelona, Spain; ^3^Departament de Medicina, Universitat Autònoma de Barcelona, Bellaterra, Spain; ^4^Hematology Department, Vall d'Hebron Hospital Universitari, Experimental Hematology, Vall d'Hebron Institute of Oncology (VHIO), Vall d'Hebron Barcelona Hospital Campus, Barcelona, Spain; ^5^Immunology Division, Vall d'Hebron University Hospital, Barcelona, Spain; ^6^Diagnostic Immunology Research Group, Vall d'Hebron Research Institute, Barcelona, Spain; ^7^Department of Cell Biology, Physiology and Immunology, Autonomous University of Barcelona (UAB), Barcelona, Spain

**Keywords:** hemophagocytic lymphohistiocytosis, cytokine storm, multiorgan dysfunction, cytokine hemoadsorption, shock

## Abstract

We discuss a single case of Hemophagocytic lymphohistiocytosis (HLH) due to NK-type non-Hodgkin lymphoma and Epstein-Barr virus reactivation with multiorgan dysfunction and distributive shock in which we performed cytokine hemoadsorption with Cytosorb ^®^. A full microbiological panel was carried out, including screening for imported disease, standard serologies and cultures for bacterial and fungal infection. A liver biopsy and bone marrow aspirate were performed, confirming the diagnosis. The patients fulfilled the HLH-2004 diagnostic criteria, and according to the 2018 Consensus Statements by the HLH Steering Committee of the Histiocyte Society, dexamethasone and etoposide were started. There was an associated hypercytokinemia and, due to refractory distributive shock, rescue therapy with cytokine hemoadsorption was performed during 24 h (within day 2 and 3 from ICU admission). After starting this procedure, rapid hemodynamic control was achieved with a significant reduction in vasopressor support requirements. This case report highlights that cytokine hemoadsorption can be an effective since rapid decrease in IL-10 levels and a significant hemodynamic improvement was achieved.

## Introduction

Hemophagocytic lymphohistiocytosis (HLH) is a severe hyperinflammatory syndrome induced by activated macrophages and cytotoxic T-cells. Secondary (acquired) HLH (1) is the most frequent form in adults, commonly triggered by infections [mainly viruses Epstein-Barr Virus (2) (EBV)], malignancies (mainly malignantlymphoma) (3), macrophage activation syndrome in autoinflammatory or autoinmmune disorders (4) or other causes (for example organ or stem cell transplantation, metabolic, traumatic, immunosupression). Untreated HLH can lead to rapid multiorgan dysfunction and death, so treatment should be started as soon as possible. The pathophysiology of organ dysfunction is led by the cytokine storm, so cytokine hemadsorption could have a potential therapeutic role in this setting.

HLH diagnosis is challenging as its clinical presentation may be indistinguishable from sepsis or autoinflammatory diseases. One of the pillars of treatment includes modulating the cytokine storm (5) responsible for hyperinflammation and multiorgan failure. Treatment should be started as promptly as possible after symptom onset as untreated HLH can lead to multiorgan failure and death. Dexamethasone, etoposide and cyclosporine A are included in the standard HLH treatment protocol (6). In addition, cytokine adsorption can be a potential therapeutical option for the rapid control of cytokine storm. Herein, we report a case of a patient with distributive shock and multiorgan dysfunction due to HLH who underwent cytokine hemoadsorption.

## Methods

We discuss a single case of HLH due to NK-type non-Hodgkin lymphoma and Epstein-Barr virus reactivation with multiorgan dysfunction and distributive shock in which we performed cytokine hemoadsorption with Cytosorb ^®^ (Cytosorbents Europe, Berlin, Germany) leading to rapid decrease in several cytokines, such as interleukin 10 (IL-10) and interleukin 6 (IL-6), with significant hemodynamic improvement. Plasmatic levels of IL-6 were measured using the automated quantitative immunoassay Cobas^®^ (Roche diagnostics International Ltd, Switzerland), following the manufacturer's instructions. Circulating levels of IL-10 and soluble CD25 (IL-2Ra) were determined using the microfluidics-based quantitative immunoassay, ELLA^®^ (ProteinSimple, United States of America). The severity of the disease was evaluated with sequential organ failure assessment (SOFA) score (7). We analyzed the plasma concentrations of inflammatory biomarkers, including IL-6, interleukin 10 (IL-10), D-dimer, and C-reactive protein upon ICU admission, immediately before hemoadsorption initiation (pre-hemoadsorption), and after the procedure (post-hemoadsorption). Other laboratory parameters were measured to evaluate organ function. The CytoSorb^®^ filter was connected post-hemofilter *via* a close loop circuit to the Continuous renal replacement therapy (CRRT) pump (Prismaflex, Gambro Lundia AB, Lund, Sweden). CRRT was performed in continuous hemodiafiltration mode (CVVHDF) using a MA 150^®^ hemofilter (Baxter, Illinois, US) at a blood flow rate of 200 ml/min. Anticoagulation was performed with unfractionated heparin.

## Results/discussion

A 50-year-old African male patient was admitted to the hospital with a 2-month history of mild epistaxis and diffuse abdominal pain. He presented fever and hepatosplenomegaly at physical exam; laboratory values showed cytolysis with undissociated cholestasis, non-oliguric renal failure, pancytopenia with hemolytic anemia, hyperferritinemia, and hypertriglyceridemia. He required a rapid admission to the Intensive Care Unit (ICU) for hemodynamic and respiratory support. A full microbiological panel was carried out, including screening for imported disease, standard serologies and cultures for bacterial and fungal infection; results came back positive for Epstein-Barr virus with a 7.7 Log viremia. The patient fulfilled the HLH-2004 diagnostic criteria, and according to the 2018 Consensus Statements by the HLH Steering Committee of the Histiocyte Society, dexamethasone and etoposide (8) were started, together with empirical antibiotic treatment with meropenem and amikacin. Simultaneously, a liver biopsy and bone marrow aspirate were performed, confirming the diagnosis of NK-type non-Hodgkin's lymphoma with secondary hemophagocytosis.

Although the patient received broad-spectrum antibiotics and the recommended HLH treatment, he rapidly deteriorated in 48 h with a distributive shock and multiorgan dysfunction (renal, neurological, hemodynamic, respiratory, and hepatic) requiring high doses of vasopressor support, continuous veno-venous hemodiafiltration and invasive mechanical ventilation. There was an associated hypercytokinemia and, due to refractory distributive shock, rescue therapy with cytokine hemoadsorption with Cytosorb^®^ was started on the second day after ICU admission. Cytokine adsorption was performed in parallel to the venovenous hemodiafiltration circuit. Interleukin 6, IL-10 and IL-2Ra were monitored in real time. After starting this procedure, rapid hemodynamic control was achieved with a significant reduction in vasopressor support requirements after only a few hours since the start of cytokine hemoadsorption (Table 1). This therapy was started within 24 hours of ICU admission. Vasopressor support was stopped 37 h after the start of this procedure. Despite this, 6 days after completion of cytokine hemoadsorption and 9 days since ICU admission, the patient died due to thrombotic complications related to the underlying lymphoma with thrombosis of the inferior vena cava, hepatic system, right atrium and partial thrombosis of the thoracic aorta.

**Table 1:** Biochemical and clinical parameters.

**Variable/Day from ICU admission**	**Day 1**	**Day 2**	**Day 3**	**Day 4**	**Day 5**	**Day 6**	**Day 7**	**Day 8**
		**Cytosorb** ^®^						
IL-10 (RV <7.8 pg/mL)	4,698	5,643	490	337	36.6	-	-	15.2
IL-6 (RV <4.3 pg/mL)	165,1	233	91,2	60,8	56,1	-	-	139.8
sCD25 (RV <2,000 pg/mL)	48,015	65,949	50,667	21,853	15,766	-	-	17,396
Ferritina (RV <400 ng/mL)	127,055	-	109,983	-	39,493	-	-	-
Bilirrubin (RV <1.2 mg/dL)	18,9	22	13	7,8	11,2	-	-	23,22
NOR (mcg/kg/min)	0	1,5	0,1	0	<0.1	<0.1	<0.1	<0.1
Lactate (RV <2 mmol/L)	4,7	8,5	10,3	5	2,1	3,7	2,3	1,7
SOFA	11	15	19	16	13	14	13	15
Creatinine (RV <1 mg/dL)	5,78	7,03	CRRT	CRRT	CRRT	CRRT	CRRT	CRRT
PAFI	-	250	286	215	262	315	272,5	300
AST (RV <50 UI/L)	926	1,938	2,626	1,625	510	376	246	108
CRP (RV <0.5 mg/dL)	8,8	7,7	5,81	4,94	-	3,3	-	8,44
Platelets (RV 140-400 x 10e9/L)	40,000	49,000	67,000	28,000	33,000	60,000	50,000	22,000

Cytosorb ^®^ therapy was done during 24 h within day 2 and 3 from ICU admission. On Day 2 we describe the worst cytokine record prior to the initiation of therapy; regarding Day 3, we reflect the variables after the completion of the hemoadsorptive therapy.AST, aspartate aminotransferase; CRRT, continuous renal replacement therapy; CRP, C-reactive protein; IL, interleukin; NOR, norepinephrine; PAFI, PaO_2_-FiO_2_ ratio; SOFA, sequential organ failure assessment; RV, Reference values.

The therapeutic strategy of HLH includes a triple approach: organ support measures, trigger resolution and inflammatory response suppression (1). Although dexamethasone, etoposide and cyclosporine A are considered standard HLH treatment, these patients present a cytokine storm that needs rapid control. The pathophysiologic process of HLH is characterized by an uncontrolled activation and expansion of T lymphocytes and macrophages, responsible for the production of a large amount of proinflammatory cytokines, the latter causing a “cytokine storm” (9). Cytokine control could be carried out with drugs such as Tocilizumab (10) or Anakinra (11) but also with extracorporeal blood purification (2).

Several extracorporeal blood purification techniques have been proposed in critical patients with multiorgan dysfunction (12). Cytosorb^®^, licensed for extracorporeal cytokine removal in the European Union, is a high-flow, low-resistance cytokine adsorbent, containing specially developed polymer beads with a large surface and adsorption spectrum up to 60kDa. Results with Cytosorb indicate not only a broad-spectrum removal of inflammatory mediators, but also significant survival improvement in high-lethality models (13).

In the sepsis cytokine-storm related pattern, IL-6 is the best studied molecule (14), but in secondary HLH the elevation of interferon gamma and IL-10 is more significant (15). Very high levels of interferon gamma and IL-10 with a mildly elevated IL-6 has a high diagnostic accuracy for secondary HLH and could be a useful approach to differentiate HLH from infection (16, 17). In this case, the indication of cytokine hemoadsorption in HLH would have as its main objective the elimination of IL-10 since the inflammatory response is unbalanced toward anti-inflammation.

Experience in the use of cytokine hemoadsorption as adjuvant treatment for HLH is scarce. Greil et al. (18) described a CTLA4-deficient patient who developed secondary HLH due to EBV-induced Hodgkin lymphoma under treatment with abatacept. Their patient also presented multiorgan dysfunction and Cytosorb^®^ was used for 4 days, achieving a reduction in inflammatory parameters and clinical improvement. This case is similar to ours, including the type of technique, since it is performed in parallel with renal hemodiafiltration. Unlike the study by Greil et al. we monitored in real time the plasma concentrations of IL-6 and IL-10 to adjust the duration of hemoadsorption. Once the cytokine levels were controlled and the patient clinically improved (with reduction in vasopressor support), we discontinued hemoadsorption. This system allows us to shorten the hemoadsorption time, an important step since it can alter the concentrations of certain drugs, such as antibiotics (19, 20). Frimmel et al. (21) reported a case of multiorgan dysfunction due to liver failure in a patient with HLH secondary to reactivation of the Herpes simplex virus type 1. In this case, the indication for hemadsorption was a distributive shock due to liver failure. In this patient, there was a very significant elevation of IL-6 but IL-10 concentrations were not reported.

As this is a case report, causality cannot be confirmed, particularly when considering the other treatment interventions (e.g., dexamethasone, etoposide). However, there was a close temporal relationship between the initiation of hemoadsorption and the reduction in cytokine levels and clinical improvement. IL-10 levels were significantly reduced in 24 h and a rapid improvement in hemodynamic dysfunction was documented 24 h from initiation of therapy, thus controlling the cytokine storm causing distributive shock. The patient's favorable clinical evolution should be attributed to all therapeutic interventions, though the rapid clinical improvement may be related to cytokine elimination by hemoadsorption, as this has not been described as a pharmacological effect of corticosteroids or etoposide. In our institution, we monitor cytokines (IL-6 and IL-10) in real time, which allows us to suspend therapy when a significant clinical and biological improvement (decrease in cytokines) has been achieved.

As we quoted before, haemadsorption therapy has been administered in parallel to conventional treatment of haemophagocytic syndrome. In our case with Etoposide and Dexamethasone. Regarding the administered dose of these immunosuppressive drugs, to date no PK data during Cytosorb is available on this treatment, so we employed conventional dosage (22). Indeed, certain pharmacokinetic consequences of hemoadsorption cannot be ruled out. However, recent studies do not show that plasma concentrations of meropenem (19) are altered, although it could be possible with other antibiotics such as teicoplanin (20). In any case, it is important to monitor antibiotic concentrations, especially when the periods of hemoadsorption are very long.

The use of haemadsorption therapy is a salvage therapy and, in this case, we indicate its use in a situation of distributive shock refractory to conventional treatment, with evolution to multi-organ dysfunction. To date, there is no threshold plasma cytokine level used for initiation or termination of therapy. In this setting, the cytokine storm in HLH is, as in sepsis, also uncontrolled and, especially in its most severe and life-threatening forms, is responsible for inflammation-driven organ damage. In this setting, blood purification techniques can blunt the inflammatory process with a rapidly considerable, nonselective effect on the cytokine storm, potentially translating into survival benefit for the patient (23).

Cytokine hemoadsorption is a safe procedure without relevant associated adverse effects (24). In our case, we did not observe adverse effects.

In our patient, cytokine hemoadsorption was associated with a rapid decrease in IL-10 levels and a significant hemodynamic improvement. This case report highlights that cytokine hemoadsorption can be an effective and safe rescue therapy in patients with HLH and multiorgan dysfunction, complementary to standard protocol treatments. We suggest real-time monitoring of plasma cytokine concentrations as a tool to monitor the biological effect of cytokine hemadsorption, optimizing the duration of this procedure.

## Data availability statement

The raw data supporting the conclusions of this article will be made available by the authors, without undue reservation.

## Ethics statement

Ethical review and approval was not required for the study on human participants in accordance with the local legislation and institutional requirements. Written informed consent to publish this case was obtained and it was recorded in the medical history. Information revealing the subject's identity was avoided.

## Author contributions

All authors carry out assistance and research work associated with the Vall d'Hebron Campus, contributed to the article, and approved the submitted version.

## Conflict of interest

The authors declare that the research was conducted in the absence of any commercial or financial relationships that could be construed as a potential conflict of interest.

## Publisher's note

All claims expressed in this article are solely those of the authors and do not necessarily represent those of their affiliated organizations, or those of the publisher, the editors and the reviewers. Any product that may be evaluated in this article, or claim that may be made by its manufacturer, is not guaranteed or endorsed by the publisher.
